# Prevalence and Patterns of Self-Reported Proton Pump Inhibitor Use Among Internal Medicine Outpatients: A Cross-Sectional Study from Türkiye

**DOI:** 10.3390/biomedicines14102193

**Published:** 2026-09-28

**Authors:** Ayşenur Uçar, Mehmet Erdevir, Felemez Arslan

**Affiliations:** 1 Department of Internal Medicine, Yüreğir State Hospital, Adana 01240, Türkiye; dr.aysenurucar@hotmail.com (A.U.); erdevirmehmet@gmail.com (M.E.); 2 Clinic of Internal Medicine, Bakırköy Dr. Sadi Konuk Training and Research Hospital, University of Health Sciences Türkiye, İstanbul 34147, Türkiye

**Keywords:** proton pump inhibitors, cross-sectional studies, outpatients, drug utilisation, deprescribing, dyspepsia, gastro-oesophageal reflux, medication review

## Abstract

**Background/Objectives:** Proton pump inhibitors (PPIs) are widely used, but real-world utilisation data from internal medicine settings remain limited. We estimated the screening-period prevalence of self-reported current PPI use among attendees of two internal medicine outpatient clinics and described treatment patterns among current users. **Methods:** In this single-centre cross-sectional survey in Adana, Türkiye (August 2025–January 2026), 977 consecutively screened adult outpatients were asked about current PPI use; 178 reported current use, and 149 of them (83.7%) completed an investigator-administered questionnaire. Wilson 95% confidence intervals (CIs) were calculated for the prevalence and three prespecified proportions; exploratory ≤6-versus > 6-month comparisons used exact tests with Holm adjustment. **Results:** Current use was reported by 178/977 outpatients (18.2%; 95% CI 15.9–20.8%). Among users (mean age 57.5 ± 13.6 years; 61.1% women), median reported duration was 54 months (interquartile range 18–84); 86.6% reported use for >6 months and 34.2% for >5 years. Pantoprazole was most common (31.5%). Gastroprotection during non-steroidal anti-inflammatory drug (NSAID) or antithrombotic therapy (45.0%) and dyspepsia (35.6%) were the leading reasons; 80.6% of those citing gastroprotection reported current NSAID or antithrombotic use. Ongoing upper-gastrointestinal symptoms were reported by 77.9% (59.7% of those citing gastroprotection; 100.0% of those citing reflux). **Conclusions:** Nearly one in five screened outpatients reported current PPI use, and reported exposure was predominantly prolonged. Ongoing symptoms and prolonged use without a documented indication are signals that should prompt structured, individualised medication review.

## 1. Introduction

Proton pump inhibitors (PPIs) are the most effective pharmacological agents for gastric acid suppression and are central to the management of gastro-oesophageal reflux disease (GERD), peptic ulcer disease, *Helicobacter pylori* eradication regimens, selected patients with dyspepsia, and gastrointestinal prophylaxis for patients at increased bleeding risk during non-steroidal anti-inflammatory drug (NSAID) or antithrombotic therapy. Treatment duration varies by indication; a limited course is sufficient for many patients, whereas maintenance therapy may be required for severe erosive oesophagitis, Barrett’s oesophagus, recurrent ulcer bleeding, or continuing high gastrointestinal bleeding risk [1,2,3].

PPI exposure is widespread, but estimates vary by population, healthcare setting, ascertainment method, and definition of long-term use. A systematic review of 65 studies from 23 countries, covering approximately 28 million PPI users, estimated that 23.4% of the adult source population were PPI users in the 28 studies that reported a source population; among the 5.3 million users contributing duration data in 22 studies, one-quarter had PPI prescriptions for one year or longer, and 27.8% of these long-term users continued for more than three years [4]. Nationwide drug-utilisation studies report annual prevalences of PPI use ranging from 7.4% of adults in Denmark and 15.5 per 100 adults in Iceland to 29.8% of adults in France, and a period prevalence of 15.0% in United Kingdom primary care [5,6,7,8]. Observational studies have associated long-term use with enteric infection, fractures, kidney disease, micronutrient deficiencies, and dementia, although residual confounding, reverse causation, surveillance bias, and exposure misclassification limit causal inference [9,10]. In a randomised trial of 17,598 participants, pantoprazole did not significantly increase most prespecified adverse outcomes over a median of 3.01 years, although enteric infections were slightly more frequent than with placebo [11].

Current guidance recommends regular documentation and reassessment of the indication, use of the lowest effective regimen, and consideration of deprescribing when no continuing indication exists. Gastrointestinal bleeding risk should be assessed before withdrawal, and discontinuation should not be based solely on duration or concern about potential adverse events [2,12,13,14]. For GERD symptom control, PPIs are generally most effective when taken 30–60 min before a meal, but administration quality cannot be determined without the dose, frequency, and interval before food [2].

In Türkiye, PPIs are prescription-only medicines. Oral PPIs are reimbursed by the Social Security Institution without a specialist-only prescribing rule or a requirement for a long-term-use report, so that they can be initiated and renewed by family physicians as well as by hospital specialists; only parenteral forms are restricted to inpatient use [15]. Utilisation is high: about 31 million PPI prescriptions were dispensed in 2012 [16], PPIs appeared in 12.8% of prescriptions issued by four family physicians in Sakarya over three years [17], and 40.8% of 103 outpatients surveyed at a training and research hospital in Uşak reported PPI use [18]. Studies of PPI users in Turkish family medicine and primary care settings found that 35.9–39.9% had no evidence-based indication and that comorbidity and polypharmacy were associated with prolonged use [19,20,21]. No Turkish study, however, has reported the prevalence of current PPI use with a complete screening denominator in an internal medicine outpatient setting.

This study aimed to estimate the screening-period prevalence of self-reported current PPI use among attendees of two internal medicine outpatient clinics and to describe current users by agent, duration, reported reasons, administration pattern, self-reported regular use, concomitant medications, and ongoing gastrointestinal symptoms. Because only current users were surveyed and the denominator comprised clinic attendees, the study describes current use in a clinical population rather than population prevalence or lifetime exposure. It was not designed to adjudicate treatment appropriateness, medication adherence, or clinical effectiveness.

## 2. Materials and Methods

### 2.1. Study Design and Setting

This single-centre, cross-sectional, investigator-administered survey was conducted in two general internal medicine outpatient clinics at Yüreğir State Hospital, a public secondary-care hospital in Adana, Türkiye. Data were collected on all working days of the two clinics between August 2025 and January 2026, after ethics approval had been obtained (Section 2.7). The clinics receive both walk-in patients and patients referred by family physicians and provide general internal medicine care for adults, predominantly follow-up of chronic diseases such as hypertension and diabetes together with dyspeptic and other general complaints. The report follows the Strengthening the Reporting of Observational Studies in Epidemiology (STROBE) recommendations for cross-sectional studies [22]; the completed checklist is provided as Supplementary Table S1.

### 2.2. Screening, Case Definition, and Participants

All adult patients (≥18 years) attending the participating clinics during the study period were approached consecutively by the investigators; 12 patients were not approached because of clinic workload. Each patient was asked a single screening question, “Şu anda mide koruyucu (pantoprazol, lansoprazol, esomeprazol, rabeprazol, omeprazol gibi) bir ilaç kullanıyor musunuz?” (“Are you currently using a stomach-protective medicine such as pantoprazole, lansoprazole, esomeprazole, rabeprazole or omeprazole?”). Current use was defined as the patient’s report of taking a PPI at the time of the visit, irrespective of the frequency of intake; regular, irregular, and on-demand users were all eligible. No minimum recent-use window was applied, and eligibility was not conditional on prescription or dispensing records. Patients attending more than once during the study period were screened only at their first attendance. Current users who provided written informed consent and completed the questionnaire formed the analytic cohort. Of 989 adult outpatients attending during the study period, 12 were not approached, and 977 were screened; 178 reported current use, none declined consent, and 29 were excluded at data entry because their paper questionnaires were incomplete or illegible, leaving 149 participants in the analytic cohort (Figure 1). On these 29 forms, most of the items, including the duration, reason-for-use and symptom sections, had not been recorded or could not be read, and only the header fields (sex and age) were consistently legible; because too few items were available to contribute meaningfully to available-case denominators, the forms were excluded as a whole. The 11 spreadsheet cells reconstructed in Section 2.5 concerned a different problem and different participants: those forms were complete and fully legible, and the cells were altered only by the spreadsheet software at data entry. The 29 non-completers were similar to the analytic cohort in sex and age (16 women, 55.2%; mean age 53.2 ± 15.0 years). No formal a priori sample-size calculation was performed; the study size was determined by the fixed data-collection period.

### 2.3. Questionnaire and Data Collection

The study-specific structured questionnaire (Supplementary File S1) was developed by the investigators on the basis of published drug-utilisation surveys and guideline documents [2,12,23] and was pilot-tested in approximately 10 patients before the study; pilot data were not included in the analysis. It was not a validated adherence or symptom scale. Before recruitment began, the two investigators agreed in a joint meeting on the wording of every question and on the coding of responses; both then administered the questionnaire face-to-face, reading each question verbatim from the same printed form and recording responses in the same predefined categories. Interviewer and clinic identifiers were not recorded on the form, so that interviewer and clinic effects cannot be assessed.

The instrument recorded age, sex, self-reported physician-diagnosed chronic conditions (open question, recorded as free text), PPI agent, duration of current use (open numeric response in months, with a supplementary weeks field for durations shorter than six months), self-reported regular use (“İlacı doktorun söylediği şekilde her gün düzenli kullanıyor musunuz?”—“Do you take the medicine regularly every day, as your doctor instructed?”; yes/no), administration in relation to meals (fasting versus with or after food) and time of day (morning, noon, evening; multiple responses allowed), reported reasons for use (six response options offered exactly as follows: gastritis; ulcer; reflux; indigestion/dyspepsia; protection of the stomach while using pain-killers or blood thinners; other), ongoing gastrointestinal symptoms (heartburn; regurgitation; epigastric pain; gas; bloating; other; none), previous upper gastrointestinal endoscopy, and current NSAID, antiplatelet, and anticoagulant therapy. Endoscopy history, reasons, and symptoms were not independently verified against medical records. In 89 of the 149 participants (59.7%), the investigators additionally reviewed the medication list of the national personal health record (e-Nabız) during the visit. These were the participants who had an e-Nabız account and were willing to share it: they displayed their own record on their own device to the investigators, who did not access any record independently of the participant. The remaining 60 participants either did not know their e-Nabız credentials or could not access the application at the visit; the subsample was therefore a convenience sample and not a random one. The information obtained (PPI agent and date of the first PPI prescription) is the same medication information that the questionnaire and the consent form cover. The recorded agent and the date of the first PPI prescription were compared with the participant’s report at the visit, and the date was considered concordant when it lay within ±3 months of the reported start of use. Only the categorical result of each comparison (agent concordant or not; date within ±3 months or not) was recorded; the recorded dates themselves were not transcribed, so the direction and size of the discrepancies cannot be analysed. Dose and prescribing specialty were not compared. Multiple reasons, symptoms, and administration times could be selected; each option was converted into a separate binary variable, and percentages could therefore exceed 100%. Participants reporting gas, bloating, or both were counted once in the participant-level gas/bloating measure.

### 2.4. Outcomes and Definitions

The primary outcome was the screening-period prevalence of self-reported current PPI use, calculated as the number of outpatients reporting current use at screening (178) divided by the number screened (977); because non-completion of the questionnaire does not alter a patient’s reported use, all users identified at screening were retained in the numerator. As a secondary, more conservative figure, the prevalence based only on users who completed the questionnaire (149/977) is also reported. Secondary outcomes were the distributions of agents, duration, reported reasons, self-reported regular use, administration pattern, ongoing symptoms, previous endoscopy, and concomitant NSAID and antithrombotic use among users. Duration was summarised continuously, dichotomised as ≤6 or >6 months (exactly six months was assigned to the shorter-duration group), and additionally categorised as ≤6, 7–12, 13–36, 37–60, and >60 months. The six-month threshold was chosen descriptively because it lies beyond the 4–12-week courses recommended for most acute indications while being less restrictive than the one-year definition of long-term use applied in the global review [2,3,4]; it was not used to define appropriateness. Fasting administration was not classified as guideline-concordant because the precise interval before food, dose, frequency, and prescribed regimen were unavailable. Treatment appropriateness, adherence, and effectiveness were not analysed. A Charlson Comorbidity Index recorded by the investigators at data entry was excluded during data preparation, before any group comparison was performed, because its version, age adjustment, component mapping, and source derivation could not be validated; the binary comorbidity variable (any reported physician-diagnosed chronic condition) was retained.

### 2.5. Data Preparation and Quality Assurance

The source workbook was preserved, and a de-identified analytic dataset was prepared with a documented analysis script. Eleven multiple-response cells had been converted to dates by the spreadsheet software at data entry: three reason-for-use cells and eight symptom cells, affecting 10 participants (one participant had both fields affected). In the workbook, multiple responses were entered as code lists separated by full stops (for example, “3.4” for codes 3 and 4); when three codes were entered (for example, “1.3.4”), the software interpreted the entry as a day.month.year date (1 March 2004). Each affected cell was therefore reconstructed deterministically as the ordered triplet {day, month, year − 2000}. The rule was accepted only when all three recovered components were valid codes, distinct, and in ascending order, which was the case for all 11 cells; ascending order is the convention observed in all 138 non-converted multiple-response cells, and none of the 11 date values could have arisen from any other code combination under the same convention. The 11 reconstructed entries were subsequently verified against the original paper questionnaires, and all matched. A sensitivity analysis nevertheless treated the 11 cells as missing. One participant recorded a duration of one month in the numeric field but “>24 weeks” in the supplementary field; the primary analysis used the numeric field, and sensitivity analyses excluded this record or reassigned it to the >6-month group. The results of all sensitivity analyses are reported in Supplementary Table S2. Missing observations were not imputed; available-case denominators were used, and comorbidity and endoscopy each had one missing value.

### 2.6. Statistical Analysis

The prevalence and three prespecified proportions among users (use for >6 months, self-reported regular use, and at least one ongoing symptom) were reported with Wilson score 95% confidence intervals (CIs); other proportions are reported as *n*/*N* (%) without intervals. Continuous distributions were assessed with histograms, Q–Q plots, and the Shapiro–Wilk test. Age was summarised as mean and standard deviation (SD); right-skewed duration was summarised as median, interquartile range (IQR), and range, and its distribution is shown in Figure 2. Digit heaping was quantified as the proportion of reported durations that were multiples of 6 and of 12 months, internal consistency was checked by calculating the implied age at initiation (age minus duration), and agreement between reported and recorded agent and start date in the e-Nabız subsample was reported as proportions with Wilson 95% CIs. Patterns by duration category and by reported reason were described without hypothesis testing. Exploratory comparisons between the ≤6- and >6-month groups used Welch’s *t* test for age and two-sided Fisher exact tests for binary variables; mean differences, prevalence ratios (PRs; Katz log-based CIs), and absolute proportion differences (Newcombe hybrid score CIs) were reported with unadjusted 95% CIs. Agent distribution across the 2 × 5 table was compared with a Monte Carlo permutation test of the χ^2^ statistic (200,000 random resamples; the Monte Carlo standard error of the *p* value is reported) and summarised with Cramér’s V. Holm adjustment covered the 10 exploratory comparisons by reported duration (Section 3.5); adjusted values are truncated at 1.00. As a sensitivity analysis for the comorbidity comparison, an age-stratified (<50, 50–64, ≥65 years) Mantel–Haenszel PR with a Greenland–Robins CI was calculated. No multivariable model was fitted because the shorter-duration group was small. Tests were two-sided with *p* < 0.05 as the conventional threshold. All reported statistics were computed in Python 3.11.15 with NumPy 2.4.4 and SciPy 1.17.1 using a documented analysis script (random seed 20260918), which is provided as Supplementary File S2; the de-identified dataset on which it runs is available from the corresponding author on reasonable request; IBM SPSS Statistics version 26 (IBM Corp., Armonk, NY, USA) was used only for data entry and initial screening.

### 2.7. Ethics

The Scientific Research Ethics Committee of Adana City Training and Research Hospital approved the study (meeting no. 16; decision no. 664; 21 August 2025). Yüreğir State Hospital does not have an institutional ethics committee; in accordance with national regulations, research conducted at public hospitals in Adana province is reviewed by this committee, which serves as the designated ethics committee for the provincial health directorate’s hospitals. Recruitment and data collection took place between August 2025 and January 2026, starting after the approval date. Written informed consent was obtained from every participant before the questionnaire was administered. The study was conducted in accordance with the Declaration of Helsinki.

## 3. Results

### 3.1. Participant Flow

Of 989 adult outpatients attending the participating clinics during the study period, 12 (1.2%) were not approached and 977 were screened (Figure 1). Current PPI use was reported by 178 (18.2%; 95% CI 15.9–20.8%) and denied by 799. None of the 178 users declined consent; 29 (16.3%) were excluded because of incomplete or illegible questionnaires (16 women; mean age 53.2 ± 15.0 years), and 149 (83.7%; 95% CI 77.6–88.4%) completed it and formed the analytic cohort. Because every non-completer had reported current use, non-completion affects the description of user characteristics but not the prevalence estimate; the estimate restricted to completed questionnaires was 15.3% (95% CI 13.1–17.6%).

**Figure 1:**
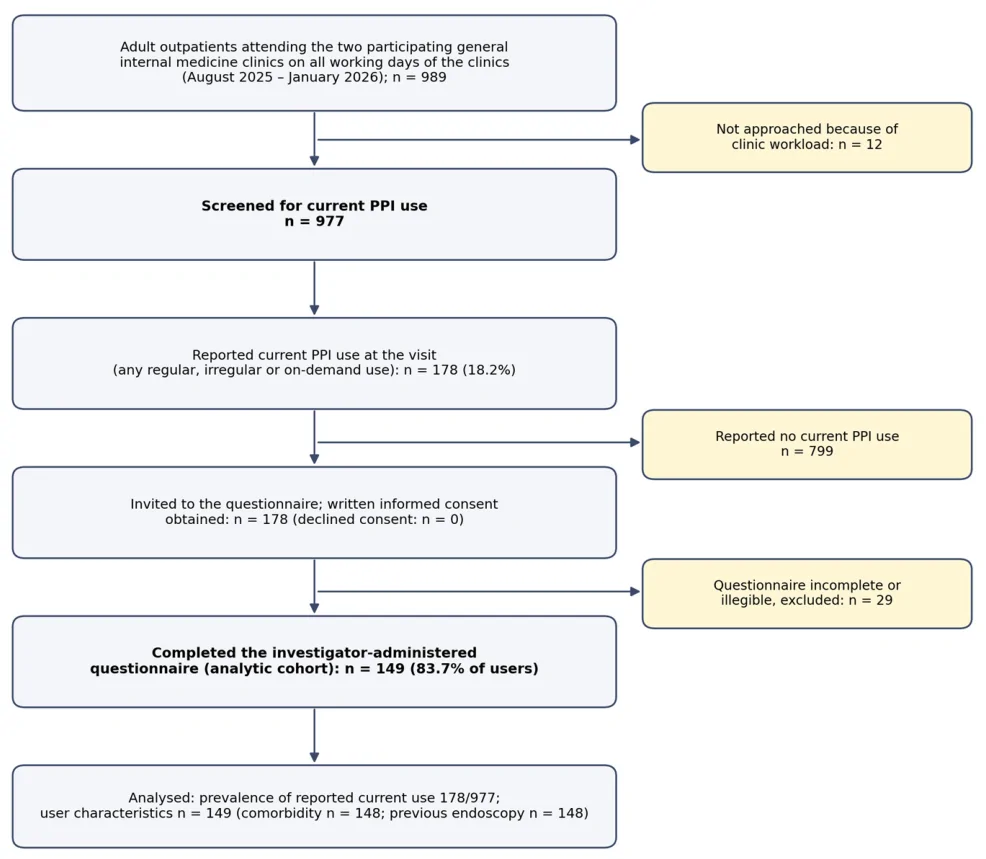
Participant flow (STROBE). Downward arrows indicate the sequence of the flow; the boxes on the right show the participants who left the flow at each step. PPI, proton pump inhibitor; STROBE, Strengthening the Reporting of Observational Studies in Epidemiology.

### 3.2. Prevalence and Characteristics of Current Users

Among the 977 screened internal medicine outpatients, 178 reported current PPI use, a screening-period prevalence of 18.2% (95% CI 15.9–20.8%) among clinic attendees; the 149 users who completed the questionnaire (83.7% of those reporting use) form the descriptive cohort. Mean age of users was 57.5 ± 13.6 years (range 18–86), and 91/149 (61.1%) were women (Table 1). Pantoprazole was the most common agent (47/149 (31.5%)), followed by lansoprazole (36/149 (24.2%)), esomeprazole (33/149 (22.1%)), rabeprazole (24/149 (16.1%)), and omeprazole (9/149 (6.0%)). Self-reported regular use was recorded for 87/149 (58.4%; 95% CI 50.4–66.0%), and 138/149 (92.6%) reported fasting administration. Morning, noon, and evening administration were reported by 139/149 (93.3%), 9/149 (6.0%), and 9/149 (6.0%) participants, respectively; eight participants selected more than one administration time. At least one comorbidity was reported by 120/148 (81.1%), most commonly hypertension (93/148 (62.8%)), diabetes mellitus (63/148 (42.6%)), coronary artery disease (31/148 (20.9%)), and hyperlipidaemia (27/148 (18.2%)) (Supplementary Table S3). Antiplatelet or anticoagulant therapy was reported by 59/149 (39.6%), NSAID use by 29/149 (19.5%), both by 7/149 (4.7%), and at least one of the two by 81/149 (54.4%); 65/148 (43.9%) reported previous upper gastrointestinal endoscopy. In the 89 participants whose e-Nabız medication list was reviewed, the recorded agent matched the reported agent in 71/89 (79.8%; 95% CI 70.3–86.8%), and the date of the first recorded PPI prescription lay within ±3 months of the reported start of use in 49/89 (55.1%; 95% CI 44.7–65.0%). Because only the categorical result of each comparison was recorded, the direction of the date discrepancies could not be determined.

### 3.3. Distribution of Reported Duration

Median reported duration was 54 months (IQR 18–84; range 1–300); 129/149 (86.6%; 95% CI 80.2–91.1%) reported use for >6 months, 84/149 (56.4%) for >3 years, 51/149 (34.2%) for >5 years, and 11/149 (7.4%) for >10 years (Figure 2). Reported values were strongly heaped: 104/129 (80.6%) of the values above 6 months were multiples of 12 months and 124/129 (96.1%) were multiples of 6 months, so that durations beyond one year should be read as approximate, self-reported estimates. All age–duration pairs were internally consistent: the lowest implied age at initiation was 17.8 years, and the two longest durations (300 months) were reported by participants aged 85 and 66 years. Characteristics by duration category are shown in Table 2. Participants reporting use for >60 months more often reported antiplatelet or anticoagulant therapy (60.8%, compared with 26.7–35.0% in the other categories) and at least one comorbidity (94.1%, compared with 55.0–85.7%). Mean age did not increase monotonically with duration: it was highest in the 7–12-month category (62.7 years), intermediate in the >60-month (60.2 years) and 37–60-month (58.8 years) categories, and lowest in the ≤6-month (53.7 years) and 13–36-month (51.6 years) categories. The proportion with at least one ongoing symptom was similar across categories (70.0–82.4%).

**Figure 2:**
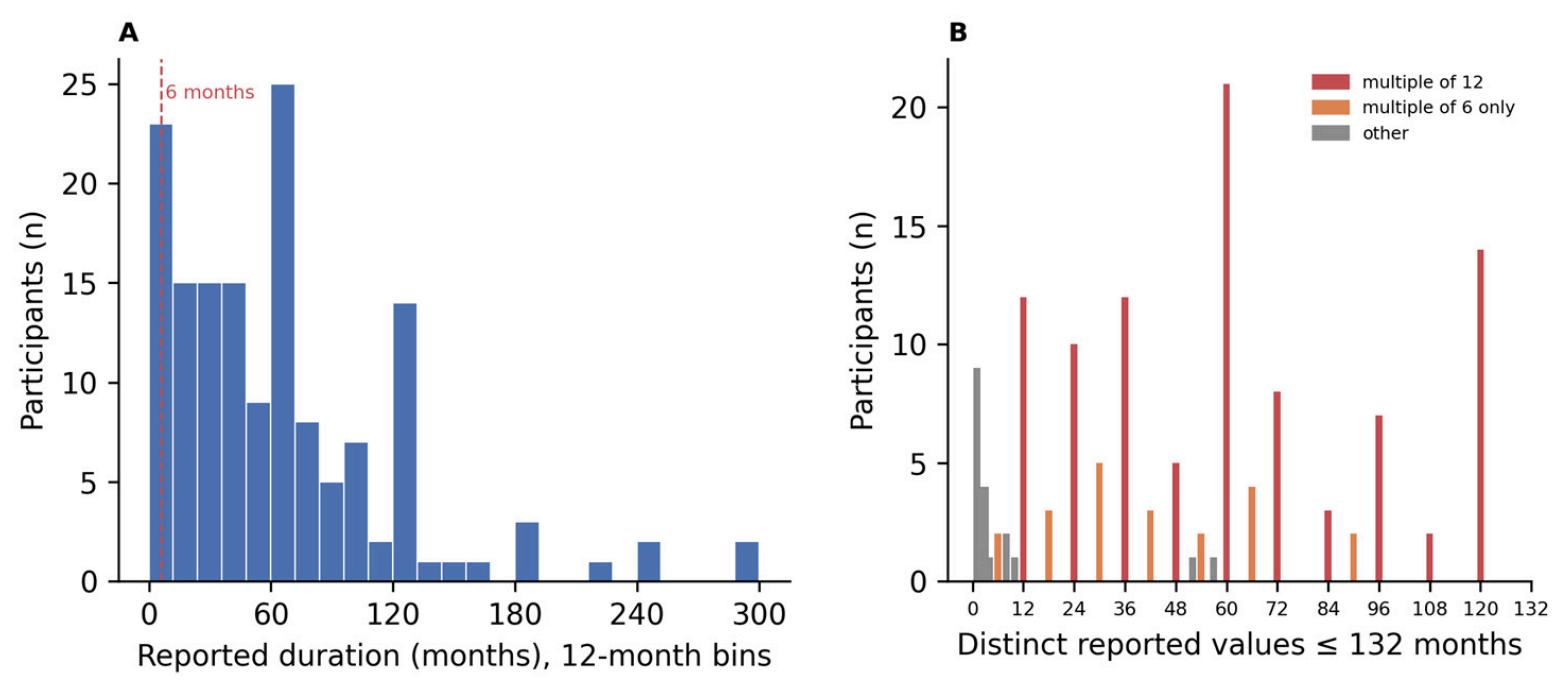
Reported duration of current proton pump inhibitor use among 149 users. (**A**) Histogram with 12-month bins; the dashed line marks 6 months. (**B**) Frequency of each distinct reported value up to 132 months, coloured by whether the value is a multiple of 12 months (red), a multiple of 6 months only (orange), or neither (grey), illustrating digit heaping.

### 3.4. Reported Reasons, Concomitant Exposure, and Ongoing Symptoms

The leading reported reasons were gastroprotection during NSAID or antithrombotic treatment (67/149 (45.0%)) and dyspepsia (53/149 (35.6%)), followed by gastritis (30/149 (20.1%)), gastro-oesophageal reflux (28/149 (18.8%)), peptic ulcer disease (11/149 (7.4%)), and other reasons (2/149 (1.3%)); 39/149 (26.2%) gave more than one reason. Of the 67 participants citing gastroprotection, 54/67 (80.6%) reported current NSAID or antithrombotic use (antithrombotic 41, NSAID 20), whereas 13/67 (19.4%) reported neither. Conversely, of the 81 participants reporting current NSAID or antithrombotic use, 54/81 (66.7%) cited gastroprotection as a reason and 27/81 (33.3%) cited only symptom- or diagnosis-based reasons. Median duration was ≥48 months for every reason category (Table 3).

At least one ongoing upper gastrointestinal symptom was reported by 116/149 (77.9%; 95% CI 70.5–83.8%), and 56/149 (37.6%) reported two or more symptoms. The proportion with ongoing symptoms varied by reason: 100.0% among those citing reflux, 96.2% among those citing dyspepsia, 86.7% among those citing gastritis, 81.8% among those citing peptic ulcer, and 59.7% among those citing gastroprotection (Table 3). Ongoing symptoms were reported by 71/87 (81.6%) of participants describing regular use and 45/62 (72.6%) of those describing irregular use, and by 106/138 (76.8%) of those reporting fasting administration and 10/11 (90.9%) of those taking the drug with or after food.

### 3.5. Exploratory Comparisons by Duration

Exploratory comparisons between participants reporting use for ≤6 months (*n* = 20) and >6 months (*n* = 129) are presented in Table 4. Self-reported regular use was less frequent in the >6-month group (56.6% versus 70.0%; PR 0.81; 95% CI 0.58–1.12; *p* = 0.33). Reported comorbidity was more frequent with >6-month use (109/128 (85.2%) versus 11/20 (55.0%); absolute difference 30.2 percentage points; 95% CI 9.7–51.6; PR 1.55; 95% CI 1.03–2.32; unadjusted *p* = 0.004; Holm-adjusted *p* = 0.04). Because the comparison is unadjusted and comorbidity was almost universal above 50 years of age (48/52 (92.3%) at 50–64 years and 49/51 (96.1%) at ≥65 years versus 23/45 (51.1%) below 50 years), an age-stratified Mantel–Haenszel PR was calculated and was attenuated (1.42; 95% CI 1.01–2.00). No other comparison approached the conventional threshold, and agent distribution did not differ between groups (χ^2^(4) = 5.97; permutation *p* = 0.19, Monte Carlo standard error 0.0009; Cramér’s V = 0.20, an imprecise estimate given 20 participants in the reference group).

### 3.6. Sensitivity Analyses

Treating the 11 reconstructed multiple-response cells as missing changed the reason and symptom frequencies by at most three participants (for example, at least one ongoing symptom 108/141 (76.6%) versus 116/149 (77.9%) in the primary analysis; the corresponding Table 4 comparison gave PR 1.11, 95% CI 0.82–1.50). Excluding the participant with the discordant duration entry (*n* = 148) or reassigning that participant to the >6-month group left every estimate in Table 4 within rounding of the primary analysis (comorbidity PR 1.62, 95% CI 1.05–2.49, and 1.62, 95% CI 1.05–2.50, respectively). Full results are given in Supplementary Table S2.

## 4. Discussion

In this single-centre cross-sectional study, 178 of 977 consecutively screened internal medicine outpatients reported current PPI use, a screening-period prevalence of 18.2% among clinic attendees, and 149 of them (83.7%) completed the questionnaire. Reported exposure was predominantly prolonged: the median duration was 4.5 years, 86.6% reported use for more than six months and one-third for more than five years. Pantoprazole was the most common agent; most participants reported morning, fasting administration, while 58.4% described regular use. Gastroprotection during NSAID or antithrombotic treatment and dyspepsia were the leading reported reasons, and 77.9% reported at least one ongoing symptom. These findings describe patterns of use; they do not establish whether treatment was appropriately indicated, adhered to, or effective.

The prevalence is a screening-period estimate among attendees of two general internal medicine clinics, and two structural features determine how it should be read. First, only current users were surveyed, so the study is silent about past use, discontinuation, and the proportion of patients ever exposed; in a Samsun family health centre survey, for instance, 60.8% of 826 attendees had ever been prescribed a PPI, whereas 213 (25.8%) had used one in the previous eight weeks [24]. Second, prevalence among internal medicine outpatients is expected to exceed population prevalence because attendance is itself associated with the conditions and co-medications for which PPIs are prescribed; the present estimate therefore over- rather than under-represents PPI use in the general population. Non-completion of the questionnaire by 29 users did not affect the estimate, because all users identified at screening were retained in the numerator. Against this background, 18.2% lies below the 40.8% reported among 103 hospital outpatients in Uşak [18], above the 15.0% period prevalence in United Kingdom primary care and the 15.5 per 100 adults in Iceland [6,8], and between the nationwide adult prevalences of Denmark (7.4%) and France (29.8%) [5,7]; clinic-based figures from other countries range from 23.2% of emergency department attendees in Spain to 31% of outpatients aged ≥40 years in Saudi Arabia [25,26]. Such comparisons remain approximate because denominators, exposure windows, and ascertainment methods differ.

The duration profile is the study’s most distinctive finding and its least verifiable measurement. Compared on the same scale, the proportion of users reporting use for more than two years (101/149 (67.8%)) exceeds the 38.4% reported among primary-care PPI users in Hatay [20]; the proportion reporting use for more than one year (114/149 (76.5%)) exceeds the 25–27% of users who continue for a year or more in prescription databases [4,8]; and the proportion reporting use for more than six months (129/149 (86.6%)) contrasts with the 4.1% of new users in France who received continuous therapy for more than six months [7]. These comparisons are approximate, because the database estimates follow cohorts of new users, whereas a survey of current users at a clinic visit over-represents people whose treatment has already lasted a long time, so that some of the excess is expected from the sampling design alone. Three features caution against over-interpretation. Reported values were heaped at multiples of 12 months (80.6% of values above six months), as expected for open recall of durations spanning years, so that the distribution beyond one year is approximate. Self-reported duration of current use also conflates continuous, intermittent, and repeatedly restarted treatment. Finally, in the subsample checked against the national personal health record, the reported agent was confirmed in 79.8% of participants, whereas the first recorded prescription fell within three months of the reported start in only 55.1%; discordance may reflect recall error, switching between agents, prescriptions predating the electronic record, or supply not captured by it, and it indicates that individual reported durations should be treated as approximate even though the overall pattern of prolonged exposure is robust. Because only the categorical result of the comparison was recorded, we cannot tell whether reported durations tended to exceed or to fall short of the recorded ones: if participants over-estimate, the median of 54 months overstates true duration; if the electronic record misses supply that predates it, it understates it. The median should therefore be read as an approximate central value, and the proportions above six, twelve and twenty-four months, which are less sensitive to errors of a few months, as the more robust description of the distribution. The agent mix (pantoprazole first, then lansoprazole and esomeprazole) matches the Hatay primary-care series [20] and differs from the esomeprazole-led Sakarya prescription data [17], suggesting regional or temporal variation in prescribing. Duration alone cannot identify inappropriate prescribing: long-term treatment may be justified for severe erosive oesophagitis, Barrett’s oesophagus, recurrent ulcer bleeding, or continuing high-risk gastroprotection [2,12,14], and reliable adjudication requires diagnosis, dose, treatment response, recurrence after withdrawal, bleeding history, and continuing risk factors, which were not available.

Reported reasons should be read in the same light. Gastroprotection during NSAID or antithrombotic treatment was cited by 45.0% of users, and the reported reason was largely concordant with reported exposure: 80.6% of those citing gastroprotection were currently taking an NSAID or antithrombotic, whereas 19.4% were not, which may reflect completed courses, prophylaxis continued after the risk drug was stopped, or misclassification. One-third of participants taking an antithrombotic or NSAID did not name gastroprotection as a reason, so some of these patients may have been protected incidentally. Exposure alone does not establish whether prophylaxis was warranted; risk stratification ordinarily considers prior ulcer or bleeding, age, NSAID dose and duration, concurrent antiplatelet, anticoagulant, or corticosteroid treatment, and comorbidity [27,28]. Dyspepsia should likewise not be classified automatically as an inappropriate reason, because guidelines support an empirical PPI trial in selected patients after appropriate *Helicobacter pylori* assessment [3]. Previous Turkish and international studies reporting potentially inappropriate use applied explicit criteria or had more detailed clinical information [19,20,29,30]; their estimates should not be transferred to this sample.

Ongoing symptoms were reported by more than three-quarters of users, and their distribution by reason is informative. Symptoms were almost universal among participants citing reflux (100.0%), dyspepsia (96.2%), or gastritis (86.7%), and much less frequent among those citing gastroprotection (59.7%), whose treatment is prophylactic rather than symptom-directed. Ongoing symptoms do not establish treatment failure; they may reflect an incompletely characterised acid-related disorder, a non-acid mechanism, an inadequate regimen, intermittent use, or inaccurate timing, and symptom severity was not measured. They nevertheless identify a large group in whom the indication, diagnosis, administration, and continued need for therapy deserve reassessment. Morning and fasting administration were reported by more than 90% of users. Both were elicited face-to-face by the treating team, and such figures are susceptible to social-desirability bias; because the interval before food, dose, and frequency were not recorded, they cannot be equated with guideline-concordant timing [2]. Similarly, a single response describing regular use is not a validated adherence measure, and intermittent or on-demand treatment may be appropriate for selected patients [2,13]. Previous endoscopy was reported by 43.9%, but endoscopy is not universally required for uncomplicated GERD or dyspepsia and is usually targeted to alarm features, risk factors, refractory symptoms, or suspected complications [2,3].

In exploratory comparisons, reported comorbidity was more frequent among participants reporting use for more than six months. We interpret this as a marker rather than an outcome: patients with chronic conditions accumulate consultations, prescriptions, and gastroprotective co-medication, so that comorbidity plausibly precedes and drives prolonged PPI use rather than the reverse, an association also described among Turkish family-medicine patients [21]. The comparison rested on 20 participants in the reference group, comorbidity was self-reported and close to a ceiling (81.1% overall), and the estimate was attenuated after age stratification; the Holm-adjusted *p* value remained below 0.05 but this single result from ten comparisons should be regarded as hypothesis-generating. The unvalidated Charlson index was excluded before comparisons were performed and was not used to strengthen the finding.

Potential harms of chronic exposure warrant attention, but much of the evidence for renal, skeletal, nutritional, or neurocognitive outcomes is observational and vulnerable to confounding [9,10], and a large randomised trial was reassuring for most measured outcomes over approximately three years while identifying a modest increase in enteric infections [11]. Our data cannot quantify appropriateness, but they indicate what a clinician or a health system could do differently. At the patient level, every consultation at which a PPI is renewed offers an opportunity to record the indication, dose, intended duration, response, and bleeding risk, and to apply a deprescribing algorithm in patients without a continuing indication, using dose reduction, on-demand use, or discontinuation, while continuing treatment in those with high-risk conditions [12,23]. At the system level, the prescription-only, reimbursed, and unrestricted status of oral PPIs in Türkiye means that renewals accumulate across prescribers without a designated point of review; the national electronic prescription system could flag continuous PPI supply beyond six or twelve months without a recorded indication, and pharmacist- or nurse-led medication review in internal medicine clinics could target the groups identified here, namely users with prolonged exposure, users with ongoing symptoms despite treatment, and users of antithrombotic or NSAID therapy without a documented gastroprotective indication.

A principal strength was the screening denominator of 977 consecutively approached outpatients, which permitted estimation of clinic-based prevalence with a confidence interval. The study also provides granular patient-reported information on agents, duration, administration, reasons, concomitant medications, comorbidity, and symptoms; reports the duration distribution and its heaping explicitly; verifies the reported agent and start of use against the national personal health record in a subsample; and uses exact tests, effect estimates with confidence intervals, multiplicity adjustment, transparent data-quality procedures, and a documented analysis script.

This study has several limitations. Its single-centre setting and clinic-based denominator limit generalisability; only current users were surveyed, and the cross-sectional design precludes temporal or causal inference. Twenty-nine of the 178 users (16.3%) were excluded because of incomplete or illegible questionnaires (Section 2.2); they remain in the prevalence numerator and were similar to the analytic cohort in age and sex, but their treatment patterns are unknown. No a priori sample-size calculation was performed, and the precision of subgroup estimates is correspondingly limited. Most measurements were self-reported and susceptible to recall, social-desirability, and exposure-misclassification bias; reported durations were heaped at annual values, and verification against the national personal health record was possible in only 60% of participants, who formed a convenience rather than a random subsample; it confirmed the agent in four-fifths of them and the reported start of use within three months in little more than half, and because only the categorical result was recorded, the direction of the discrepancies is unknown. Dose, frequency, precise administration interval, prescriber, initial indication, renewal history, treatment response, *Helicobacter pylori* status, symptom severity, and complete bleeding-risk variables were unavailable. The questionnaire was study-specific, pilot-tested only in a small group, and not a validated adherence or symptom instrument, and interviewer and clinic effects could not be assessed because identifiers were not recorded. Comorbidity was ascertained by an open question and was not clinically adjudicated. The six-month threshold was descriptive, subgroup analyses were exploratory and underpowered, and comorbidity and endoscopy each had one missing observation. Eleven multiple-response cells required deterministic reconstruction after spreadsheet auto-formatting; the reconstructed entries were verified against the original paper forms and sensitivity analyses were reassuring. These limitations prevent reliable adjudication of appropriateness, adherence, or effectiveness.

## 5. Conclusions

Nearly one in five consecutively screened internal medicine outpatients reported current PPI use, and reported exposure among users was predominantly prolonged, with a median of 4.5 years and one-third exceeding five years. Ongoing upper gastrointestinal symptoms were reported by most users, particularly those treated for symptom-based indications, and one-third of patients taking antithrombotic or NSAID therapy did not identify gastroprotection as a reason for treatment. These patterns do not measure inappropriate use, but they identify groups in whom systematic documentation and periodic, individualised reassessment of indication, dose, duration, response, and gastrointestinal bleeding risk are most likely to be worthwhile.

## Figures and Tables

**Table 1:** Prevalence of current proton pump inhibitor use and characteristics of current users.

Characteristic	*n*/*N* (%)
Reported current PPI use among screened outpatients ^a^	178/977 (18.2); 95% CI 15.9–20.8%
Users completing the questionnaire (analytic cohort)/prevalence based on completed questionnaires	149/178 (83.7)/149/977 (15.3); 95% CI 13.1–17.6%
Age, years, mean (SD); median (IQR); range	57.5 (13.6); 57 (47–67); 18–86
Female sex	91/149 (61.1)
Duration of current use, months, median (IQR); range	54 (18–84); 1–300
Duration >6 months ^a^	129/149 (86.6); 95% CI 80.2–91.1%
Duration >12 months/>36 months/>60 months	114/149 (76.5)/84/149 (56.4)/51/149 (34.2)
Agent: pantoprazole/lansoprazole/esomeprazole	47/149 (31.5)/36/149 (24.2)/33/149 (22.1)
Agent: rabeprazole/omeprazole	24/149 (16.1)/9/149 (6.0)
Self-reported regular use ^a^	87/149 (58.4); 95% CI 50.4–66.0%
Fasting administration/with or after food	138/149 (92.6)/11/149 (7.4)
Administration time: morning/noon/evening ^b^	139/149 (93.3)/9/149 (6.0)/9/149 (6.0)
Reason: gastroprotection during NSAID or antithrombotic therapy ^b^	67/149 (45.0)
Reason: dyspepsia (indigestion) ^b^	53/149 (35.6)
Reason: gastritis ^b^	30/149 (20.1)
Reason: gastro-oesophageal reflux ^b^	28/149 (18.8)
Reason: peptic ulcer/other ^b^	11/149 (7.4)/2/149 (1.3)
At least one ongoing symptom ^a^	116/149 (77.9); 95% CI 70.5–83.8%
Symptom: epigastric pain ^b^	55/149 (36.9)
Symptom: gas and/or bloating (participant-level union) ^b^	53/149 (35.6)
Symptom: regurgitation/heartburn/other ^b^	29/149 (19.5)/16/149 (10.7)/23/149 (15.4)
At least one reported comorbidity ^c^	120/148 (81.1)
Antiplatelet and/or anticoagulant therapy	59/149 (39.6)
Current NSAID use/both/either exposure	29/149 (19.5)/7/149 (4.7)/81/149 (54.4)
Previous upper gastrointestinal endoscopy ^c^	65/148 (43.9)

Data are *n*/*N* (%) unless stated otherwise. ^a^ Wilson 95% confidence intervals (CIs) are given only for the prevalence and the three prespecified proportions. ^b^ Multiple responses were allowed; percentages may exceed 100%. ^c^ Comorbidity and endoscopy were each missing for one participant. IQR, interquartile range; NSAID, non-steroidal anti-inflammatory drug; PPI, proton pump inhibitor; SD, standard deviation.

**Table 2:** Characteristics of current users by category of reported duration.

Characteristic	≤6 Months (*n* = 20)	7–12 Months (*n* = 15)	13–36 Months (*n* = 30)	37–60 Months (*n* = 33)	>60 Months (*n* = 51)
Age, years, mean (SD)	53.7 (17.0)	62.7 (14.1)	51.6 (12.6)	58.8 (13.8)	60.2 (11.2)
Female sex	12/20 (60.0)	11/15 (73.3)	18/30 (60.0)	24/33 (72.7)	26/51 (51.0)
Self-reported regular use	14/20 (70.0)	8/15 (53.3)	16/30 (53.3)	15/33 (45.5)	34/51 (66.7)
At least one ongoing symptom	14/20 (70.0)	11/15 (73.3)	24/30 (80.0)	25/33 (75.8)	42/51 (82.4)
Reason: gastroprotection	8/20 (40.0)	9/15 (60.0)	8/30 (26.7)	18/33 (54.5)	24/51 (47.1)
Reason: dyspepsia	5/20 (25.0)	7/15 (46.7)	12/30 (40.0)	12/33 (36.4)	17/51 (33.3)
At least one comorbidity ^a^	11/20 (55.0)	12/14 (85.7)	23/30 (76.7)	26/33 (78.8)	48/51 (94.1)
Antiplatelet and/or anticoagulant therapy	7/20 (35.0)	4/15 (26.7)	8/30 (26.7)	9/33 (27.3)	31/51 (60.8)
Current NSAID use	5/20 (25.0)	6/15 (40.0)	4/30 (13.3)	9/33 (27.3)	5/51 (9.8)
Previous endoscopy ^a^	8/20 (40.0)	8/15 (53.3)	14/30 (46.7)	12/32 (37.5)	23/51 (45.1)
Most common agent	pantoprazole (7)	pantoprazole (6)	pantoprazole (11)	lansoprazole (11)	esomeprazole (13)

Data are *n*/*N* (%) unless stated otherwise; descriptive only, no hypothesis tests were performed across categories. ^a^ Available-case denominators. NSAID, non-steroidal anti-inflammatory drug; SD, standard deviation.

**Table 3:** Reported duration and ongoing symptoms by reported reason for proton pump inhibitor use.

Reported Reason ^a^	*n*	Duration, Months, Median (IQR)	>36 Months	≥1 Ongoing Symptom	Epigastric Pain	Gas/Bloating	Regurgitation	Heartburn
Gastroprotection during NSAID/antithrombotic therapy	67	60 (15–78)	42/67 (62.7)	40/67 (59.7)	12/67 (17.9)	17/67 (25.4)	6/67 (9.0)	2/67 (3.0)
Dyspepsia	53	48 (18–84)	29/53 (54.7)	51/53 (96.2)	26/53 (49.1)	35/53 (66.0)	13/53 (24.5)	8/53 (15.1)
Gastritis	30	60 (36–96)	21/30 (70.0)	26/30 (86.7)	19/30 (63.3)	11/30 (36.7)	5/30 (16.7)	4/30 (13.3)
Gastro-oesophageal reflux	28	48 (24–60)	16/28 (57.1)	28/28 (100.0)	8/28 (28.6)	9/28 (32.1)	13/28 (46.4)	7/28 (25.0)
Peptic ulcer disease	11	72 (39–114)	8/11 (72.7)	9/11 (81.8)	6/11 (54.5)	5/11 (45.5)	2/11 (18.2)	1/11 (9.1)
Other	2	36; 120 ^b^	1/2 (50.0)	2/2 (100.0)	2/2 (100.0)	0/2 (0.0)	2/2 (100.0)	0/2 (0.0)

^a^ Multiple reasons could be selected (39 participants gave more than one reason); rows are therefore not mutually exclusive. ^b^ The two individual values are shown because a median and interquartile range are not meaningful for *n* = 2. Data are *n*/*N* (%) unless stated otherwise; descriptive only. IQR, interquartile range; NSAID, non-steroidal anti-inflammatory drug.

**Table 4:** Exploratory comparisons of current users by reported duration of proton pump inhibitor use (≤6 versus >6 months).

Characteristic	≤6 Months (*n* = 20)	>6 Months (*n* = 129)	Effect, >6 vs. ≤6 Months (95% CI)	*p*	Holm-Adjusted *p* ^a^
Age, years, mean (SD)	53.7 (17.0)	58.1 (13.0)	MD 4.42 (−3.78 to 12.63)	0.28	1.00
Female sex	12/20 (60.0)	79/129 (61.2)	PR 1.02 (0.70–1.50)	1.00	1.00
PPI-agent distribution ^b^	—	—	Cramér’s V 0.20	0.19	1.00
Self-reported regular use	14/20 (70.0)	73/129 (56.6)	PR 0.81 (0.58–1.12)	0.33	1.00
Fasting administration	19/20 (95.0)	119/129 (92.2)	PR 0.97 (0.87–1.09)	1.00	1.00
At least one ongoing symptom	14/20 (70.0)	102/129 (79.1)	PR 1.13 (0.84–1.53)	0.39	1.00
At least one comorbidity ^c^	11/20 (55.0)	109/128 (85.2)	PR 1.55 (1.03–2.32); Δ 30.2 pp (9.7–51.6)	0.004	0.04
Antiplatelet and/or anticoagulant therapy	7/20 (35.0)	52/129 (40.3)	PR 1.15 (0.61–2.17)	0.81	1.00
Current NSAID use	5/20 (25.0)	24/129 (18.6)	PR 0.74 (0.32–1.72)	0.55	1.00
Previous endoscopy ^c^	8/20 (40.0)	57/128 (44.5)	PR 1.11 (0.63–1.97)	0.81	1.00

Age: Welch’s *t* test; binary variables: two-sided Fisher exact tests; effect-estimate CIs are unadjusted (Katz log method for PRs; Newcombe hybrid score method for the absolute difference). ^a^ Holm adjustment covered the 10 comparisons in this table; adjusted values are truncated at 1.00. ^b^ Exact permutation test of the 2 × 5 table: χ^2^(4) = 5.97, 200,000 Monte Carlo resamples, Monte Carlo standard error of *p* = 0.0009; agent counts (≤6/>6 months): pantoprazole 7/40, lansoprazole 5/31, esomeprazole 1/32, rabeprazole 6/18, omeprazole 1/8. ^c^ Available-case denominators. CI, confidence interval; MD, mean difference; NSAID, non-steroidal anti-inflammatory drug; PPI, proton pump inhibitor; PR, prevalence ratio; pp, percentage points; SD, standard deviation.

## Data Availability

The analysis script that reproduces every statistic in this article, together with the log of the reconstructed cells and a README describing the software environment, is provided as Supplementary File S2; it runs directly on the de-identified participant-level dataset, which is available from the corresponding author on reasonable request. The source workbook contains identifying information and cannot be shared.

## Supplementary Materials

The following supporting information can be downloaded at: https://www.mdpi.com/article/10.3390/biomedicines14102193/s1, Supplementary File S1: Study questionnaire; Table S1: STROBE checklist for cross-sectional studies; Table S2: Sensitivity analyses and log of reconstructed multiple-response cells; Table S3: Self-reported comorbid conditions among current users; Supplementary File S2: Analysis script.
