# Supplementary material for: Prevalence and Patterns of Self-Reported Proton Pump Inhibitor Use Among Internal Medicine Outpatients: A Cross-Sectional Study from Türkiye
Source: Biomedicines. 2026 Sep 28;14(10):2193. doi: 10.3390/biomedicines14102193 (PMC13701674; doi:10.3390/biomedicines14102193)
Supplement: Supplementary file 1 [file biomedicines-14-02193-s001.zip › PPI_Supplementary_Materials_published_numbering.pdf]

Supplementary Materials

Prevalence and Patterns of Self-Reported Proton Pump Inhibitor Use Among Internal Medicine Outpatients: A Cross-Sectional Study from Türkiye

Ayşenur Uçar, Mehmet Erdevir and Felemez Arslan

Supplementary File S1. Study questionnaire

S1.1 English translation of the investigator-administered questionnaire. The questionnaire was developed by the investigators on the basis of published drug-utilisation surveys and guideline documents, pilot-tested in approximately 10 patients (not included in the analysis), and administered face to face by the two investigators, who read each question verbatim and recorded the responses on the printed form. Turkish wording is given for the screening and regular-use items; the Turkish original of the full form is provided in S1.2.

No.	Question (as read to the patient)	Response options / coding
S	Screening question: "Şu anda mide koruyucu (pantoprazol, lansoprazol, esomeprazol, rabeprazol, omeprazol gibi) bir ilaç kullanıyor musunuz?" — "Are you currently using a stomach-protective medicine such as pantoprazole, lansoprazole, esomeprazole, rabeprazole or omeprazole?"	Yes → eligible (any regular, irregular or on-demand use); No → not eligible (counted in the screening denominator)
1	Age	Years (integer)
2	Sex	Female / Male
3	Which proton pump inhibitor are you using? (name and strength)	Pantoprazole; lansoprazole; esomeprazole; rabeprazole; omeprazole; strength (mg) if known
4	For how long have you been using this medicine?	Open numeric answer in months; for durations shorter than six months, weeks were additionally recorded
5	"İlacı doktorun söylediği şekilde her gün düzenli kullanıyor musunuz?" — "Do you take the medicine regularly every day, as your doctor instructed?"	1 = yes; 0 = no
6	How do you take it in relation to meals?	1 = on an empty stomach (before food); 2 = with or after food
7	At what time of day do you take it?	1 = morning; 2 = noon; 3 = evening (more than one answer allowed)
8	Why do you use this medicine?	1 = gastritis; 2 = ulcer; 3 = reflux; 4 = indigestion/dyspepsia; 5 = to protect the stomach while using pain-killers or blood thinners; 6 = other (more than one answer allowed)
9	Do you currently have any of the following complaints?	1 = heartburn; 2 = regurgitation; 3 = epigastric pain; 4 = gas; 5 = bloating; 6 = other; 0 = none (more than one answer allowed)
10	Do you have any chronic disease diagnosed by a physician? Which?	Free text (e.g., hypertension, diabetes mellitus, coronary artery disease, …); coded as any = 1 / none = 0
11	Have you ever had an upper gastrointestinal endoscopy (gastroscopy)?	1 = yes; 0 = no
12	Are you currently taking a blood thinner (antiplatelet or anticoagulant)?	1 = yes; 0 = no
13	Are you currently taking a non-steroidal anti-inflammatory pain-killer?	1 = yes; 0 = no

S1.2 Turkish original. Wording of the printed questionnaire administered in the study, reproduced in typeset form; item numbering as in S1.1.

İç Hastalıkları Polikliniği'nde Proton Pompa İnhibitörü Kullanımı: Sıklık, Tedaviye Uyum, Etkinlik
Hasta Anket Formu

Araştırmacı tarafından yüz yüze uygulanır; sorular hastaya okunur ve yanıtlar bu forma kaydedilir.

Tarih: ____ / ____ / ______ Dosya No: ______________ Ad Soyad: _________________________

Tarama sorusu: Şu anda mide koruyucu (pantoprazol, lansoprazol, esomeprazol, rabeprazol, omeprazol gibi) bir ilaç kullanıyor musunuz?

☐ Evet → ankete devam edilir ☐ Hayır → anket sonlandırılır (tarama sayısına dahil edilir)

1. Yaşınız?

______ yıl

2. Cinsiyetiniz?

☐ Kadın ☐ Erkek

3. Hangi mide koruyucu ilacı kullanıyorsunuz? (ilacın adı ve dozu)

☐ Pantoprazol ☐ Lansoprazol ☐ Esomeprazol ☐ Rabeprazol ☐ Omeprazol Doz (mg, biliniyorsa): ______

4. Bu ilacı ne zamandır kullanıyorsunuz?

______ ay (6 aydan kısa ise: ______ hafta)

5. İlacı doktorun söylediği şekilde her gün düzenli kullanıyor musunuz?

☐ Evet (1) ☐ Hayır (0)

6. İlacı nasıl alıyorsunuz?

☐ Aç karnına, yemekten önce (1) ☐ Tok karnına, yemekle birlikte veya yemekten sonra (2)

7. İlacı günün hangi zamanında alıyorsunuz? *(birden fazla seçenek işaretlenebilir)*

☐ Sabah (1) ☐ Öğlen (2) ☐ Akşam (3)

8. Bu ilacı neden kullanıyorsunuz? *(birden fazla seçenek işaretlenebilir)*

☐ Gastrit (1) ☐ Ülser (2) ☐ Reflü (3) ☐ Hazımsızlık (4) ☐ Ağrı kesici veya kan sulandırıcı kullanırken mideyi korumak için (5) ☐ Diğer (6): ______

9. Şu anda aşağıdaki şikâyetlerden herhangi biri var mı? *(birden fazla seçenek işaretlenebilir)*

☐ Mide yanması (1) ☐ Ağza acı/ekşi su gelmesi (2) ☐ Mide (karın üst bölgesi) ağrısı (3) ☐ Gaz (4) ☐ Şişkinlik (5) ☐ Diğer (6): ______ ☐ Yok (0)

10. Doktor tarafından tanı konmuş kronik bir hastalığınız var mı? Hangisi/hangileri?

☐ Yok (0) ☐ Var (1): ______________________

11. Daha önce hiç mide endoskopisi (gastroskopi) yaptırdınız mı?

☐ Evet (1) ☐ Hayır (0)

12. Şu anda kan sulandırıcı bir ilaç (antiagregan veya antikoagülan) kullanıyor musunuz?

☐ Evet (1) ☐ Hayır (0)

13. Şu anda steroid olmayan anti-inflamatuar bir ağrı kesici (NSAİİ) kullanıyor musunuz?

☐ Evet (1) ☐ Hayır (0)

Araştırmacının notu: ____

Table S1. STROBE checklist for cross-sectional studies

Item	No.	Recommendation	Location in the article
Title and abstract	1	(a) Indicate the study design in the title or abstract; (b) informative and balanced abstract	Title ("A Cross-Sectional Study"); Abstract
Background/rationale	2	Scientific background and rationale	Section 1, paragraphs 1–4
Objectives	3	State specific objectives	Section 1, last paragraph
Study design	4	Key elements of study design	Section 2.1
Setting	5	Setting, locations, dates of recruitment and data collection	Section 2.1 (August 2025 – January 2026)
Participants	6	Eligibility criteria, sources and methods of selection	Section 2.2; Figure 1
Variables	7	Outcomes, exposures, predictors, potential confounders	Sections 2.3–2.4
Data sources/measurement	8	Sources of data and methods of assessment	Section 2.3; Supplementary File S1
Bias	9	Efforts to address potential sources of bias	Sections 2.3, 2.5; Discussion (limitations)
Study size	10	How the study size was arrived at	Section 2.2
Quantitative variables	11	Handling of quantitative variables; groupings	Section 2.4
Statistical methods	12	(a) methods; (b) subgroups/interactions; (c) missing data; (d) sampling strategy; (e) sensitivity analyses	Sections 2.5–2.6; Table S2
Participants	13	Numbers at each stage; reasons for non-participation; flow diagram	Section 3.1; Figure 1
Descriptive data	14	Characteristics; missing data per variable	Section 3.2; Table 1; Table S3
Outcome data	15	Numbers of outcome events or summary measures	Sections 3.2–3.4; Tables 1–3; Figure 2
Main results	16	Unadjusted and adjusted estimates with precision; category boundaries	Sections 3.2, 3.5; Tables 1 and 4
Other analyses	17	Subgroups, interactions, sensitivity analyses	Sections 3.3–3.6; Table S2
Key results	18	Summarise key results with reference to objectives	Section 4, paragraph 1
Limitations	19	Limitations, sources of bias, direction and magnitude	Section 4, last paragraph
Interpretation	20	Cautious overall interpretation	Section 4; Section 5
Generalisability	21	External validity	Section 4, paragraph 2; limitations
Funding	22	Source of funding and role of funders	Back matter (Funding)

Table S2. Sensitivity analyses and log of reconstructed multiple-response cells

S2.1 Log of the 11 spreadsheet cells auto-converted to dates and their deterministic reconstruction

Participant (de-identified sequential no.)	Field	Cell value as stored	Recovered codes (day, month, year – 2000)	Valid, distinct, ascending
2	Ongoing symptoms	2004-03-01	1, 3, 4	yes
20	Reason for use	2005-04-03	3, 4, 5	yes
27	Ongoing symptoms	2006-04-03	3, 4, 6	yes
38	Ongoing symptoms	2004-03-02	2, 3, 4	yes
43	Ongoing symptoms	2003-02-01	1, 2, 3	yes
45	Reason for use	2004-03-01	1, 3, 4	yes
45	Ongoing symptoms	2005-02-01	1, 2, 5	yes
97	Ongoing symptoms	2004-03-02	2, 3, 4	yes
126	Ongoing symptoms	2004-03-01	1, 3, 4	yes
132	Ongoing symptoms	2005-04-03	3, 4, 5	yes
143	Reason for use	2004-03-01	1, 3, 4	yes

Reason codes: 1 gastritis; 2 peptic ulcer; 3 gastro-oesophageal reflux; 4 dyspepsia; 5 gastroprotection; 6 other. Symptom codes: 1 heartburn; 2 regurgitation; 3 epigastric pain; 4 gas; 5 bloating; 6 other. Three-code entries typed as "d.m.y" (e.g., "1.3.4") were stored by the spreadsheet as dates (1 March 2004). All 138 non-converted multiple-response cells in the workbook were typed in ascending order, and all 11 recovered triplets are valid, distinct and ascending; the 11 reconstructed entries were subsequently verified against the original paper questionnaires and all matched.

S2.2 Sensitivity analysis A: the 11 reconstructed cells treated as missing

Variable	Primary analysis, n/149 (%)	Sensitivity A, n/N (%)
Reason: gastroprotection	67/149 (45.0)	66/146 (45.2)
Reason: dyspepsia	53/149 (35.6)	50/146 (34.2)
Reason: gastritis	30/149 (20.1)	28/146 (19.2)
Reason: gastro-oesophageal reflux	28/149 (18.8)	25/146 (17.1)
Reason: peptic ulcer	11/149 (7.4)	11/146 (7.5)
Reason: other	2/149 (1.3)	2/146 (1.4)
Symptom: epigastric pain	55/149 (36.9)	48/141 (34.0)
Symptom: regurgitation	29/149 (19.5)	25/141 (17.7)
Symptom: heartburn	16/149 (10.7)	12/141 (8.5)
Symptom: other	23/149 (15.4)	22/141 (15.6)
Symptom: gas and/or bloating	53/149 (35.6)	46/141 (32.6)
At least one ongoing symptom	116/149 (77.9)	108/141 (76.6)
Table 4 row "≥1 ongoing symptom", >6 vs ≤6 months	102/129 (79.1) vs 14/20 (70.0); PR 1.13 (0.84–1.53); p = 0.39	94/121 (77.7) vs 14/20 (70.0); PR 1.11 (0.82–1.50); p = 0.57

S2.3 Sensitivity analyses B and C: the participant with discordant duration entries (numeric field 1 month; supplementary field ">24 weeks")

Table 4 comparison	Primary (n = 149)	B: record excluded (n = 148)	C: record reassigned to >6 months (n = 149)
Female sex	PR 1.02 (0.70–1.50); p = 1.00; Holm p = 1.00	PR 0.97 (0.67–1.40); p = 1.00; Holm p = 1.00	PR 0.96 (0.66–1.39); p = 1.00; Holm p = 1.00
Self-reported regular use	PR 0.81 (0.58–1.12); p = 0.33; Holm p = 1.00	PR 0.83 (0.59–1.16); p = 0.46; Holm p = 1.00	PR 0.83 (0.59–1.17); p = 0.46; Holm p = 1.00
Fasting administration	PR 0.97 (0.87–1.09); p = 1.00; Holm p = 1.00	PR 0.97 (0.87–1.09); p = 1.00; Holm p = 1.00	PR 0.97 (0.87–1.10); p = 1.00; Holm p = 1.00
≥1 ongoing symptom	PR 1.13 (0.84–1.53); p = 0.39; Holm p = 1.00	PR 1.16 (0.84–1.59); p = 0.37; Holm p = 1.00	PR 1.16 (0.84–1.59); p = 0.37; Holm p = 1.00
≥1 comorbidity	PR 1.55 (1.03–2.32); p = 0.004; Holm p = 0.04	PR 1.62 (1.05–2.49); p = 0.002; Holm p = 0.02	PR 1.62 (1.05–2.50); p = 0.002; Holm p = 0.02
Antiplatelet/anticoagulant	PR 1.15 (0.61–2.17); p = 0.81; Holm p = 1.00	PR 1.09 (0.59–2.04); p = 1.00; Holm p = 1.00	PR 1.09 (0.58–2.03); p = 1.00; Holm p = 1.00
Current NSAID use	PR 0.74 (0.32–1.72); p = 0.55; Holm p = 1.00	PR 0.71 (0.31–1.63); p = 0.53; Holm p = 1.00	PR 0.70 (0.30–1.62); p = 0.53; Holm p = 1.00
Previous endoscopy	PR 1.11 (0.63–1.97); p = 0.81; Holm p = 1.00	PR 1.06 (0.60–1.85); p = 1.00; Holm p = 1.00	PR 1.05 (0.60–1.84); p = 1.00; Holm p = 1.00

Table 4 comparison	Primary (n = 149)	B: record excluded (n = 148)	C: record reassigned to >6 months (n = 149)
Age, years (Welch t test)	MD 4.42 (-3.78 to 12.63); p = 0.28	MD 5.49 (-2.82 to 13.81); p = 0.18	MD 5.61 (-2.70 to 13.93); p = 0.17
PPI-agent distribution	$\chi^2(4) = 5.97$; permutation p = 0.19; V = 0.20	$\chi^2(4) = 6.07$; permutation p = 0.19; V = 0.20	$\chi^2(4) = 6.10$; permutation p = 0.19; V = 0.20

PR, prevalence ratio (>6 vs ≤6 months) with Katz 95% CI; MD, mean difference; Holm adjustment over the 10 comparisons of Table 4, truncated at 1.00. Under the age-stratified (<50, 50–64, ≥65 years) Mantel–Haenszel analysis of the comorbidity comparison in the primary dataset, the PR was 1.42 (95% CI 1.01–2.00); stratum-specific counts (>6 vs ≤6 months): <50 years 22/36 vs 1/9; 50–64 years 45/48 vs 3/4; ≥65 years 42/44 vs 7/7.

Table S3. Self-reported physician-diagnosed comorbid conditions among current users (n = 148 with available data)

Condition (as recorded)	n/148 (%)
Hypertension (HT)	93/148 (62.8)
Diabetes mellitus (DM)	63/148 (42.6)
Coronary artery disease (KAH)	31/148 (20.9)
Hyperlipidaemia (HL)	27/148 (18.2)
Asthma (ASTIM)	11/148 (7.4)
Cerebrovascular disease (stroke) (SVO)	9/148 (6.1)
Chronic obstructive pulmonary disease (KOAH)	4/148 (2.7)
Hypothyroidism (HİPOTİROİDİ)	4/148 (2.7)
Breast cancer (MEME CA)	3/148 (2.0)
Heart failure (KKY)	3/148 (2.0)
Chronic kidney disease (KBH)	2/148 (1.4)
Aortic stenosis (AS)	2/148 (1.4)
Hypothyroidism (variant spelling) (HIPOTIROIDI)	1/148 (0.7)
Liver cirrhosis (KC S)	1/148 (0.7)
Immune thrombocytopenia (ITP)	1/148 (0.7)
Nasopharyngeal cancer (NAZOFARENKS CA)	1/148 (0.7)
Adrenal cancer (ADRENAL BEZ CA)	1/148 (0.7)
Bladder cancer (MESANE CA)	1/148 (0.7)
Polycythaemia vera (PV)	1/148 (0.7)
Cardiac condition under cardiology follow-up (not further specified) (KT)	1/148 (0.7)
Rheumatoid arthritis (RA)	1/148 (0.7)

Number of conditions per participant: 0 = 28; 1 = 36; 2 = 42; 3 = 28; 4 = 13; 5 = 1. Conditions were elicited by an open question and recorded as free text by the investigators; any reported physician-diagnosed condition qualified for the binary comorbidity variable. Hypothyroidism is shown as two spelling variants in the source and counts as one condition (5 participants). A Charlson Comorbidity Index recorded at data entry (median 2, interquartile range 1–4) was excluded from the analysis because its derivation could not be validated.

Supplementary File S2. Analysis script

The Python script that produces every statistic, table value and Figure 2 in the article (PPI_analysis_script.py) is supplied as a separate file with this Supplementary Material, together with the log of the 11 reconstructed multiple-response cells (PPI_reconstructed_cells_log.json) and a README describing the software environment (Python 3.11, NumPy 2.4.4, SciPy 1.17.1; random seed 20260918). The script runs directly on the de-identified analytic dataset (`python3 PPI_analysis_script.py PPI_analytic_dataset_deidentified.csv`) and reproduces the results file used for the manuscript; running it in this mode reproduces every reported statistic (the only quantity not reproduced is the summary of the recorded Charlson Comorbidity Index, which was excluded from the analysis and is not part of the de-identified dataset). The de-identified dataset is available from the corresponding author on reasonable request.
